# Willingness to pay for HPV vaccine among female health care workers in a Chinese nationwide survey

**DOI:** 10.1186/s12913-022-08716-6

**Published:** 2022-11-05

**Authors:** Xinyue Lu, Mengdi Ji, Abram L. Wagner, Weifeng Huang, Xiaoping Shao, Weiyu Zhou, Yihan Lu

**Affiliations:** 1 Department of Epidemiology, Ministry of Education Key Laboratory of Public Health Safety, Fudan University School of Public Health, 131 Dong An Road, 200032 Shanghai, China; 2 Department of Epidemiology, University of Michigan, 48109 Ann Arbor, MI USA; 3 Department of Intensive Care Medicine, The Sixth People’s Hospital, Shanghai Jiao Tong University, 200233 Shanghai, China; 4 131 Dong An Road, 200032 Shanghai, China

**Keywords:** Human papillomavirus vaccine, HPV, Willingness to pay, WTP, Contingent valuation method, Female health care workers, China

## Abstract

**Background:**

Payment methods for human papillomavirus (HPV) vaccine could substantially influence vaccination behavior. In China, HPV vaccination uptake remains currently low. This study aims to determine willingness to pay (WTP) for HPV vaccines among Chinese female health care workers under different payment scenarios.

**Methods:**

This is a nationwide online survey recruiting female health care workers aged 18–45 years from 31 provinces throughout China. We collected the respondents’ vaccination status of HPV vaccines and their sociodemographics. Two WTPs were defined and estimated in the study. A general WTP for HPV vaccination was determined using the contingent valuation method with double dichotomous choice bidding. A WTP out-of-pocket was estimated for each HPV vaccine under two scenarios, including partial coverage by governmental subsidy or partial incorporation in basic medical insurance. Accordingly, a multivariable linear regression model was employed to determine the association between sociodemographis and general WTP. Then the maximum WTP out-of-pocket was compared among the respondents’ attitude shift towards HPV vaccination, payment scenarios, and levels of vaccine attributes, using non-parametric Kruskal-Wallis test.

**Results:**

A total of 15,969 respondents were included in the study. The median general WTP was 2000 CNY (interquartile range, 1000–3200 CNY), positively associated with younger age, unmarried status, higher monthly income, fewer children, more positive vaccination behavior, working in tertiary hospital, higher local GDP and HDI (each *P* < 0.05). Moreover, the median WTP out-of-pocket was 1250 CNY (540–2000 CNY). It was significantly higher for vaccines partly covered by governmental subsidy (median, 1250 CNY; interquartile range, 560–2000 CNY), imported vaccines (1260 CNY; 630–1960 CNY), and 9-valent vaccines (1400 CNY; 750–2240 CNY) (each *P* < 0.001). Additionally, majority of respondents did not change their attitude towards HPV vaccination between two payment scenarios; those remaining with more expensive HPV vaccines (51.1%) had higher WTP out-of-pocket (1400 CNY; 560–2250 CNY) than those with cheaper vaccines (39.0%) (1120 CNY; 490–1960 CNY) (*P* < 0.001).

**Conclusion:**

Chinese female health care workers have high WTP for HPV vaccines. A direct public funding for HPV vaccination is more preferable. Our findings may facilitate the adjustment of HPV vaccination strategy and payment mechanism in China.

**Supplementary Information:**

The online version contains supplementary material available at 10.1186/s12913-022-08716-6.

## Introduction

Human papillomavirus (HPV) is the most common sexually transmitted infection [1], with the probability of HPV infection exceeding 80% for women and 90% for males across their lifetime [2]. HPV can cause anogenital warts, recurrent respiratory papillomatosis, oropharyngeal cancer, and a variety of anogenital cancers in men and women [3]. Cervical cancer is the fourth most frequently diagnosed cancer and the fourth leading cause of cancer death in women, with an estimated 604,000 new cases and 342,000 deaths worldwide in 2020. In China, there are an estimated 110,900 new cases and 59,000 deaths from cervical cancer according to nationwide cancer statistics by the National Cancer Center in 2020 [4]. The World Health Organization (WHO) called for all countries to take action to help end the suffering caused by cervical cancer in May 2018 [5]. In addition, WHO Global Action Plan for the Prevention and Control of Noncommunicable Diseases 2013–2020 identifies HPV vaccination and cervical cancer screening and treatment as best buys. These interventions have been further recommended for inclusion in WHO member states’ national health plans [6].

HPV vaccination has been proven effective in the prevention of cervical cancer in both high-income countries and low- and middle-income countries [7]. However, it has been estimated that cervical cancer burden may continually increase in the future 15 years in countries like China and Japan which have low uptake of the vaccine [8]. In China, there are routine physical examination programs or certain research programs for HPV screening, whereas no national screening program. Moreover, HPV vaccination uptake remains currently low due to multiple factors, including limited supplies of HPV vaccines, low awareness of the vaccination, and concern of possible adverse events following immunization [9]. Moreover, vaccine price could substantially influence vaccination behavior, as has been found for the influenza vaccine in the UK, rotavirus vaccine in Asian countries, and HPV vaccine in those countries with highest burden of cervical cancer [10–12]. The cost-effectiveness of HPV vaccination remains inconsistent among different studies, which might be partly attributable to the high price of HPV vaccines [13]. Thus, there warrants further improvement in the pricing and payment mechanism for increasing the uptake of HPV vaccines. This is particularly important in countries or localities which are deciding whether to provide direct public funding for vaccinations, or instead rely on coverage through insurance programs.

Since 2020, multiple cities have included pilot HPV immunization programs in China’s mainland. Diverse strategies have been implemented, such as governmental subsidies and payment by basic medical insurance for certain or non-certain HPV vaccines [14]. It has been documented that different payment mechanisms may influence the willingness to pay (WTP) and vaccine uptake, such as COVID-19 vaccine in China [15]. There are usually two common methods for estimating WTP using the theory of random utility, including discrete choice experiment and contingent valuation method [16–18]. These methods have been utilized in identifying the acceptable price thresholds for dengue vaccine and pneumococcal vaccine [19–22]. In addition, female health care workers have more professional knowledge and make decisions on HPV vaccination depending more on their own knowledge and awareness, compared with general female population. Thus, this study was performed to assess the WTP for HPV vaccines under two payment scenarios among female health care workers aged 18–45 and then identify the factors that could potentially influence the WTP. The findings would facilitate further improvement of HPV vaccination strategies in Chinese adult females.

## Materials and methods

### Study design

We performed a cross-sectional study to investigate the female health care workers on HPV vaccination behavior and their WTP for HPV vaccines. Using an online questionnaire with convenience sampling method, a total of 15,969 female health care workers aged 18 to 45 years were recruited from 31 provinces in China’s mainland between November 15 and December 22, 2021. In the questionnaire, sociodemographics and HPV vaccination behavior were collected. Moreover, WTP was determined for HPV vaccines, including four actual vaccines (domestic 2-valent, imported 2-valent, imported 4-valent, and imported 9-valent) and one assumed vaccine (domestic 9-valent), under two payment scenarios (partly covered by governmental subsidy or partly incorporated in basic medical insurance).

In addition, we included the Human Development Index (HDI), which is a long-standing summary measure developed by the United Nations Development Programme (UNDP) [23], to assess the impact of development in different regions on the WTP for HPV vaccines. The HDI is defined as the average achievement of the following three dimensions of human development: a long and healthy life (based on life expectancy at birth); being knowledgeable (based on mean and expected years of schooling); and having a decent standard of living (based on gross national income per capita). The HDI was assessed according to four levels (low, < 0.55; medium, 0.55–0.7; high, 0.7–0.8; very high, ≥ 0.8).

### HPV vaccination behavior

We investigated the respondents’ vaccination status of HPV vaccines and prepared four responses, including “had been vaccinated”, “had not been vaccinated but had made an appointment”, “had not been vaccinated or made an appointment but intend to receive vaccination”, and “not intend to receive vaccination”.

Subsequently, the first two responses were collapsed together, because they had a positive vaccination behavior. We classified these responses into three groups, “had been vaccinated or had made an appointment” (positive), “had not been vaccinated or made an appointment” (neutral), and “not intend to vaccinate” (negative). In a multivariable linear regression model, we scored three groups as follows: 3 points for “had been vaccinated or had made an appointment”, 2 points for “had not been vaccinated or made an appointment”, and 1 point for “not intend to vaccinate”.

### WTP measurement

Using the contingent valuation method, a combination of closed and open-ended questions was used to precisely determine the respondents’ general WTP, which was defined as a WTP for general HPV vaccination rather than certain vaccines. In the double dichotomous choice bidding format, we prepared questions on two stages. In the initial stage, the questions contained a range of bid amounts (800 CNY, 1600 CNY, 2400 CNY, 3200 CNY, and 4000 CNY), to which respondents stated their WTP as “yes” or “no” to hypothetical vaccine price. These initial bid amounts were randomly distributed among respondents during the questionnaire survey. In the follow-up stage, the questions were listed with bid amount of a 400 CNY plus or minus to the initial bid amounts depending on the response to the initial bid amounts. If the response to the initial bid amount was “yes”, follow-up bid amount increased by a 400 CNY; if the response to the initial bid amount was “no”, follow-up bid amount decreased by a 400 CNY. For example, in the initial stage, a question was asked to respondents, such as “Are you willing to pay for the HPV vaccine of 4000 CNY, regardless of your HPV vaccination status?”. If respondents answered “yes” to the initial bid amount, follow-up bid amount was redirected to be 4400 CNY in the follow-up stage; if response answered “no” to the initial bid amount, follow-up bid amount was redirected to be 3600 CNY.

Consequently, responses to the double-bounded contingent valuation questions were classified as four dichotomous outcomes: (1) respondents were not willing to pay for HPV vaccines at both initial bid amount and a lower follow-up bid amount (“no”, “no”); (2) respondents were not willing to pay for HPV vaccines at the initial bid amount but willing to pay at a lower follow-up bid amount (“no”, “yes”); (3) respondents were willing to pay for HPV vaccines at the initial bid amount but not at a higher follow-up bid amount (“yes”, “no”); and (4) respondents were willing to pay for HPV vaccines at both the initial bid amount and a higher follow-up bid amount (“yes”, “yes”). These four dichotomous outcomes were used as a categorical variable in the analysis. Furthermore, for all respondents, an open-ended question was prepared to ask for the maximum amount of general WTP, which was considered as a continuous variable in the analysis.

In addition, we prepared two questions to investigate the minimum percentage of cost of an HPV vaccine under two payment scenarios, for which respondents are willing to pay the remaining cost of vaccine (“if governmental subsidy or basic medical insurance could cover partial cost of HPV vaccine, which minimum percentage of the coverage do you expect when you are willing to pay the remaining cost”). We utilized a 9-item Likert scale, consecutively from 10% to 90% with an incremental percentage of 10%. Respondents’ maximum WTP out-of-pocket was calculated for each HPV vaccine, including four actual and one assumed vaccines, based on their actual and estimated prices and expected minimum percentage covered by governmental subsidy or basic medical insurance.

### Statistical analysis

Descriptive statistics were presented. Four dichotomous responses of the general WTP (“no”, “no”; “no”, “yes”; “yes”, “no”; “yes”, “yes”) were compared across the sociodemographics using chi-square tests. Then a multivariable linear regression model was employed to determine the association between explanatory variables and general WTP [24, 25]. The variance inflation factor (VIF) [26], White test [27], and Kolmogorov-Smirnov test [28] were used to assess the multicollinearity, heteroscedasticity, and residual normality of the data, respectively. A VIF value of lower than 10 was used to define no multicollinearity between variables. A *P* value greater than 0.05 in the White test and Kolmogorov-Smirnov test indicated no heteroscedasticity and normal distribution of residuals, respectively. Explanatory variables that were not significant at *P* < 0.05 in the initial model were excluded. The final model was achieved with stepwise regression method using EViews 9.0 (IHS Global INC, Irvine, CA).

Respondents’ attitudes toward HPV vaccines between two payment scenarios were compared across sociodemographics using chi-square tests. Then the maximum WTP out-of-pocket was compared among the attitude shift, payment scenarios, and levels of vaccine attributes. As the maximum WTP out-of-pocket was non-normally distributed continuous variable, we utilized non-parametric Kruskal-Wallis test. All the analyses were conducted using SAS version 9.4 (SAS Institute, Cary, NC). Significance was assessed at *P* < 0.05.

## Results

### Respondents’ sociodemographics

A total of 15,969 female health care workers aged 18–45 years from 31 provinces in China’s mainland completed the questionnaire. The mean age of the respondents was 30.6 ± 6.2 years, with 4822 (30.2%) aged 18–26 years, 7714 (48.3%) aged 27–35 years, and 3433 (21.5%) aged 36–45 years. Majority of the respondents were married (61.1%), Han Chinese (93.3%), had a bachelor degree (68.3%), monthly income less than 8000 CNY (82.3%), primary professional title (57.7%), worked in tertiary hospitals (74.2%), lived in the city with GDP of ≥ 1.6 trillion CNY (42.4%) and HDI of 0.7–0.8 (63.0%).

### General WTP for HPV vaccination

In the contingent valuation questions, the percentage of respondents whose responses to initial and follow-up bid amount were “yes, yes” gradually decreased with increasing initial bid amount, while those whose responses were “no, no” gradually increased (Table 1). When the initial amount was 800 CNY, 1600 CNY, 2400 CNY and 3200 CNY, the percentage of respondents whose responses were “yes, yes” was the highest (66.2%, 57.1%, 51.1%, and 40.8%, respectively); in contrast, when the initial amount was 4000 CNY, the percentage of those whose responses were “no, no” was the highest (47.6%) (Table 1). Furthermore, in each group with diverse initial bid amounts, respondents aged 18–26, with monthly income > 8000 CNY, no child, and positive vaccination behavior were more likely to choose “yes, yes” (each *P* < 0.05), whereas those aged 36–45, married, living in a city with a GDP ≤ 0.6 trillion CNY, with middle professional title or above, and who did not intend to vaccinate were more likely to choose “no, no” (each *P* < 0.05) (Supplementary Table 1).

**Table 1:** Number of respondents stratified by willingness to pay for HPV vaccines

Initial bid amounts (CNY) (*N* = 15,969)	Initial bid response	Follow-up bid amounts (CNY)	Follow-up bid response	Number of respondents (%)
800 (*N* = 3393)	No	400	No	182 (5.7)
			Yes	204 (6.4)
	Yes	1200	No	694 (21.7)
			Yes	2113 (66.2)
1600 (*N* = 3394)	No	1200	No	605 (19.0)
			Yes	278 (8.7)
	Yes	2000	No	487 (15.2)
			Yes	1824 (57.1)
2400 (*N* = 3394)	No	2000	No	868 (27.2)
			Yes	280 (8.8)
	Yes	2800	No	411 (12.9)
			Yes	1635 (51.1)
3200 (*N* = 3394)	No	2800	No	1198 (37.5)
			Yes	334 (10.5)
	Yes	3600	No	357 (11.2)
			Yes	1305 (40.8)
4000 (*N* = 3394)	No	3600	No	1520 (47.6)
			Yes	281 (8.8)
	Yes	4400	No	275 (8.6)
			Yes	1118 (35.0)

Moreover, the median general WTP was determined to be 2000 CNY (interquartile range, IQR, 1000–3200 CNY) for HPV vaccination. A multivariable linear regression model indicated that respondents who were younger, unmarried, had a higher monthly income, fewer children, positive vaccination behavior, worked in tertiary hospitals, and lived in the cities with higher GDP and HDI, would intend to pay higher WTP (Table 2), which was similar to the findings when the general WTP was classified as four dichotomous responses.

**Table 2:** Factors associated with willingness to pay for HPV vaccines among Chinese female health workers

Variables	Initial model					Final model				
	Coefficient	Std. Error	t-Statistic	*P* value	Centered VIF	Coefficient	Std. Error	t-Statistic	*P* value	Centered VIF
Constant	1201.70	85.43	14.07	*< 0.001*	-	1646.63	77.15	21.34	*< 0.001*	-
Age	-223.82	21.86	-10.24	***< 0.001***	2.28	-220.02	19.94	-11.03	***< 0.001***	1.84
Educational level	19.15	22.78	0.84	*0.401*	1.19	-	-	-	*-*	-
Marital status	-202.87	33.46	-6.06	***< 0.001***	2.43	-170.07	33.57	-5.07	***< 0.001***	2.42
Local GDP	28.90	14.28	2.02	***0.040***	1.42	35.36	14.31	2.47	***0.008***	1.41
Local HDI	54.16	24.81	2.18	***0.031***	1.44	58.62	24.93	2.35	***0.012***	1.43
Professional title	-22.86	22.89	-1.00	*0.317*	1.77	-	-	-	*-*	-
Hospital level	104.22	24.38	4.27	***< 0.001***	1.06	96.94	24.55	3.95	***< 0.001***	1.04
Monthly income (CNY)	191.72	14.77	12.98	***< 0.001***	1.13	183.82	14.89	12.34	***< 0.001***	1.12
Numbers of children	-95.00	21.53	-4.41	***< 0.001***	2.35	-96.18	22.03	-4.37	***< 0.001***	2.28
Vaccination behavior	326.46	24.94	13.13	***< 0.001***	1.15	288.75	24.53	11.77	***< 0.001***	1.13

Weighted statistics (initial model vs. final model) were determined as follows: R-squared (0.95 vs. 0.95), adjusted R-squared (0.93 vs. 0.94 ), S.E. of regression (1314.94 vs. 1332.48), sum squared resid (2.77E + 10 vs. 2.83E + 10), log likelihood (-137317.4 vs. -137530.5), F-statistic (139.65 vs. 151.68), probability (F-statistic) (< 0.001 vs. < 0.001), mean dependent var (1987.48 vs. 2184.60), S.D. dependent var (1130.41 vs. 1389.13), Akaike info criterion (17.20 vs. 17.23), Schwarz criterion (17.21 vs. 17.23), Hannan-Quinn criter (17.21 vs. 17.23), Durbin-Watson stat (1.96 vs. 1.98)

### WTP out-of-pocket under two payment scenarios

We classified the respondents’ attitude shift towards HPV vaccines between two payment scenarios (partly covered by governmental subsidy and by basic medical insurance). In the respondents, 51.1% and 39.0% intended to remain with more expensive HPV vaccines and remain with cheaper vaccines, respectively, regardless of payment scenario. Few respondents changed the attitudes, including choosing more expensive vaccines (7.2%) or cheaper vaccines (2.7%) if governmental subsidy is available instead of basic medical insurance. Respondents differed significantly by their attitude shift towards HPV vaccines across sociodemographics (Supplementary Table 2).

The median WTP out-of-pocket was calculated to be 1250 CNY (IQR, 540–2000) and significantly differed among the attitude shift (*P* < 0.001). It was lowest (median, 1120 CNY; IQR, 490–1960 CNY) in those remaining with cheaper vaccine regardless of payment scenario, while highest (median, 1400 CNY; IQR, 560–2250 CNY) in those remaining with more expensive vaccine regardless of payment scenario (Table 3).

**Table 3:** Willingness to pay out-of-pocket for HPV vaccines by attitude shift between two payment scenarios

Attitude shift towards HPV vaccines	Number of respondents (%)	Willingness to pay (CNY)	
		Median	Interquartile range
Respondents remaining with cheaper vaccine, regardless of payment scenario	6222 (39.0)	1120	490, 1960
Respondents choosing more expensive vaccine if partly covered by governmental subsidy (instead of basic medical insurance)	1148 (7.2)	1250	500, 2000
Respondents moving from more expensive to cheaper vaccine if partly covered by governmental subsidy (instead of basic medical insurance)	433 (2.7)	1250	500, 2000
Respondents remaining with more expensive vaccine, regardless of payment scenario	8166 (51.1)	1400	560, 2250

### WTP out-of-pocket associated with vaccine attributes

**Fig. 1:**
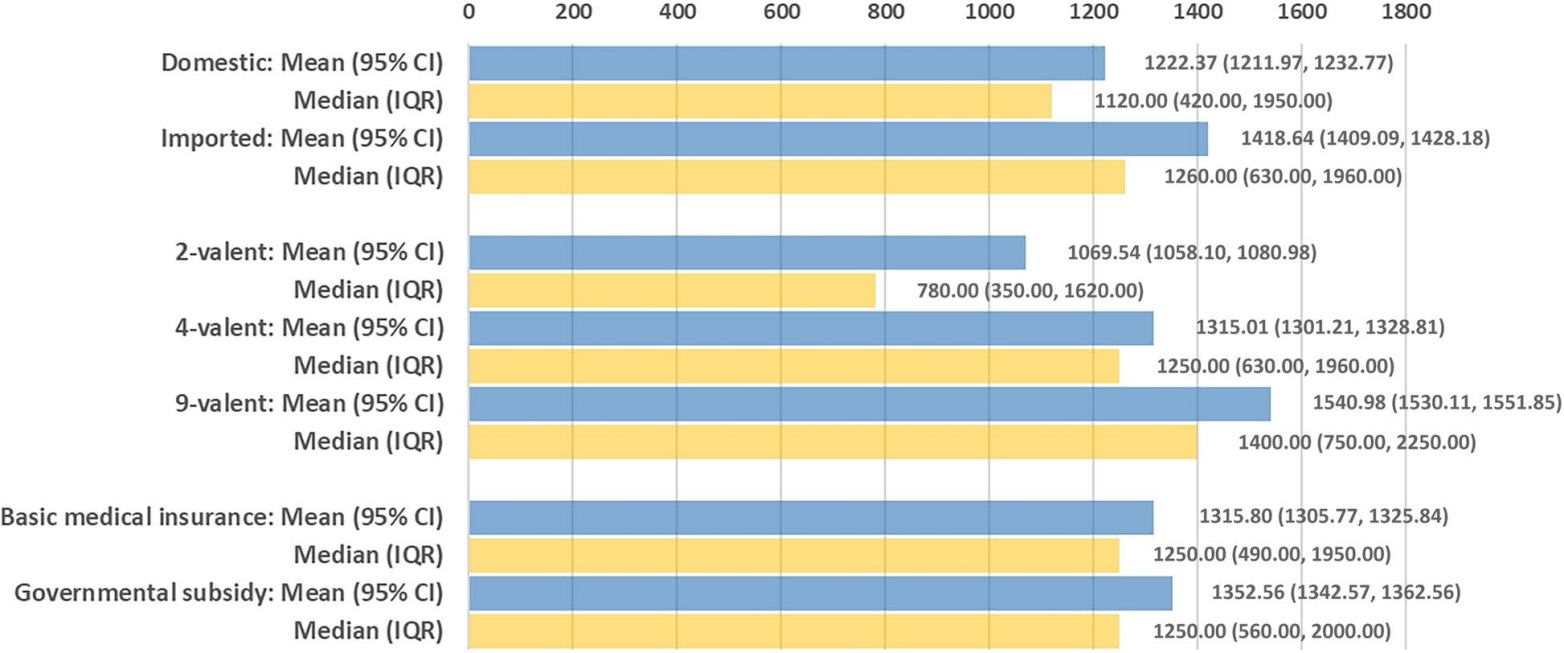
Maximum willingness to pay (WTP) out-of-pocket for an HPV vaccine, stratified by vaccine attributes, including vaccine manufacturer (domestic and imported), valency of vaccine (2-valent, 4-valent and 9-valent), and payment scenario (partly covered by governmental subsidy and by basic medical insurance)

We further determined the mean WTP out-of-pocket according to vaccine attributes, including vaccine manufacturer (domestic and imported), valency of vaccine (2-valent, 4-valent and 9-valent), and payment scenario (governmental subsidy and basic medical insurance). Respondents preferred to pay higher WTP out-of-pocket if governmental subsidy is available for each HPV vaccine (each *P* < 0.05) (Table 4). Remarkably, median WTP was approximately 50% of the current actual and estimated prices of each HPV vaccine, regardless of payment scenario. The WTP out-of-pocket was significantly higher for imported vaccines (median, 1260 CNY; IQR, 630–1960 CNY), 9-valent vaccines (median, 1400 CNY; IQR, 750–2240 CNY), and HPV vaccines partly covered by governmental subsidy (median, 1250 CNY; IQR, 560–2000 CNY) (each *P* < 0.001) (Fig. 1).

**Table 4:** Willingness to pay out-of-pocket for HPV vaccines under two payment scenarios

HPV vaccines	Price of HPV vaccine (CNY)	Partly covered by basic medical insurance		Partly covered by governmental subsidy		*P value*
		Mean (SD)	Median (IQR)	Mean (SD)	Median (IQR)	
2-valent, domestic	700 (actual)	385.29 (185.27)	350 (280, 560)	395.11 (182.76)	350 (280, 560)	*0.007*
2-valent, imported	1800 (actual)	955.44 (497.47)	900 (540, 1440)	1043.81 (468.33)	900 (900, 1440)	*0.005*
4-valent, imported	2500 (actual)	1400.44 (667.24)	1250 (1000, 2000)	1442.90 (646.50)	1250 (1250, 2000)	*< 0.001*
9-valent, domestic	2800 (estimated)	1582.28 (749.19)	1400 (1120, 2240)	1624.98 (723.53)	1400 (1400, 2240)	*0.001*
9-valent, imported	3900 (actual)	2189.51 (1057.43)	1950 (1560, 3120)	2255.99 (1023.42)	1950 (1950, 3120)	*< 0.001*

## Discussion

Our study determined the WTP for HPV vaccines among female health care workers in a nationwide survey in China’s mainland. The median general WTP and WTP out-of-pocket was 2000 CNY (approximately 303 USD) and 1250 CNY (approximately 189 USD), respectively. As a female professional population, their WTP was moderately higher than the 49 USD which was documented in women in Vietnam in 2018 [29]. However, WTP out-of-pocket was determined to be half of the current actual and estimated prices for each HPV vaccine, suggesting it remains crucial to reduce vaccine prices or improve payment mechanisms for increasing vaccine uptake.

We found several demographic characteristics were strongly related to higher WTP for the HPV vaccines. HPV vaccination should preferably be performed in younger females to have the most impact, so respondents who were older, married, and had children in this study intended to pay a lower general WTP. Similar findings were also found in previous studies in Vietnam and Hong Kong [29, 30]. Notably, our study included local GDP and HDI as variables in the analysis and identified a positive association with WTP, suggesting that both individual and regional socioeconomic status may increase WTP for HPV vaccines. In a study in the United States in 2020, socioeconomic factors had a strong influence on the HPV vaccination behavior [31]. These studies reveal the need to improve payment mechanisms, particularly in areas of the country which are more socioeconomically disadvantaged.

We found the payment mechanism significantly impacted WTP. Specifically, if HPV vaccines are partly covered by governmental subsidy, WTP out-of-pocket was significantly higher across all five HPV vaccines, compared to if vaccines are partly incorporated in basic medical insurance. It might be interpreted that respondents believe the payment for HPV vaccines by basic medical insurance would cost their own money in the medical insurance that would have paid for more medicine, while payment by governmental subsidy is kind of direct additional support. This finding may provide a clue to design more appropriate payment mechanism for HPV vaccination. In addition, WTP out-of-pocket was highest among respondents remaining with more expensive HPV vaccines, whereas lowest among those remaining with cheaper vaccines, regardless of payment scenario. It indicated the WTP varied along with choice of vaccines, though vaccine price had limited effect on the choice decision, which was also observed in parents of middle school students towards HPV vaccination in China [32]. Currently, multiple cities in China’s mainland have established diverse payment mechanisms for expanding HPV vaccination, including by basic medical insurance and by governmental subsidy [33]. Therefore, it is urgently necessary to prospectively observe the change in the HPV vaccination intent and subsequent uptake in these cities.

In addition, our study explored the difference in the WTP associated with vaccine manufacturer and valency. We found that the WTP for 9-valent vaccine was higher than those for 4-valent and 2-valent (1400 CNY vs. 1250 CNY and 780 CNY). Similarly, a study in 2020 found that Chinese parents of primary school students were also willing to pay more for 9-valent HPV vaccines, rather than 4-valent or 2-valent [9]. However, the WTP for imported HPV vaccines was higher than that for domestic vaccines (1260 CNY vs. 1120 CNY) in our study, which was similar to previous findings that imported HPV vaccines were highly preferable in China [34]. Furthermore, our study included an assumed 9-valent domestic vaccine in the analysis, in addition to four actual vaccines. It may suggest there is a need of a multi-valent domestic HPV vaccine with a lower price for expanding HPV vaccination in China. Therefore, our findings may provide new insights into the adjustment of HPV vaccination strategy and preparation of a more proper payment mechanism.

Limitations of this study need to be discussed. First, we recruited female health care workers in 31 provinces throughout China’s mainland, which may improve the representativeness and generalizability of our study. However, there could be biases due to the socioeconomic disparity across the nation. Second, in applying contingent valuation techniques, respondents could have been influenced by the range of values chosen for the payment scale question design rather than their true maximum WTP values. It is also possible that some respondents may have stated no WTP value or very low WTP value, especially if they feel that HPV vaccination should be paid by the government.

Our study has several strengths. First, with our large sample size and robust findings, our study provides empirical evidence on the WTP for HPV vaccines among Chinese female health care workers. Second, we defined and calculated two WTPs in the study. A general WTP determined the cost for HPV vaccines that respondents could afford, and a WTP out-of-pocket determined the amount that respondents willing to pay for each vaccine under either a governmental subsidy or coverage in basic medical insurance. These WTPs may comprehensively reflect the respondents’ intent to pay for HPV vaccines and their payment preference.

## Conclusion

This study found that Chinese female health care workers had a high general WTP for HPV vaccines, which was associated with sociodemographics and local GDP and HDI. When comparing scenarios of payment that is covered by governmental subsidy vs. incorporation in basic medical insurance, 90.1% of respondents did not change their attitudes towards HPV vaccination. Moreover, they preferred to pay higher WTP out-of-pocket for vaccines partly covered by governmental subsidy, imported vaccines, and 9-valent vaccines. Our study suggests a direct public funding for HPV vaccination may be more preferable among Chinese female health care workers.

## Supplementary Information

**Additional file 1:** **Supplementary Table 1.** Response to diverse initial bid amounts across sociodemographics among Chinese female health care workers. **Supplementary Table 2.** Attitude shift towards HPVvaccines between two payment scenarios across sociodemographics.

## Data Availability

The datasets used and/or analysed during the current study are available from the corresponding author Dr. Yihan Lu on reasonable request.

## Abbreviations

CNY: Chinese Yuan
COVID-19: Coronavirus Disease 2019
GDP: Gross Domestic Product
HDI: Human Development Index
HPV: Human papillomavirus
IQR: Interquartile range
USD: United States dollar
WHO: World Health Organization
WTP: Willingness to pay
