# Supplementary material for: Willingness to pay for HPV vaccine among female health care workers in a Chinese nationwide survey
Source: BMC Health Serv Res. 2022 Nov 5;22:1324. doi: 10.1186/s12913-022-08716-6 (PMC9637298; doi:10.1186/s12913-022-08716-6)
Supplement: Supplementary file 1 — Additional file 1: Supplementary Table 1. Response to diverse initial bid amounts across sociodemographics among Chinese female health care workers. Supplementary Table 2. Attitude shift towards HPVvaccines between two payment scenarios across sociodemographics. [file 12913_2022_8716_MOESM1_ESM.docx]

**Supplementary Table 1. Response to diverse initial bid amounts across sociodemographics among Chinese female health care workers**

|  | Initial bid amount: 800 CNY | | | | Initial bid amount: 1600 CNY | | | | Initial bid amount: 2400 CNY | | | | Initial bid amount: 3200 CNY | | | | Initial bid amount: 4000 CNY | | | |
| --- | --- | --- | --- | --- | --- | --- | --- | --- | --- | --- | --- | --- | --- | --- | --- | --- | --- | --- | --- | --- |
|  | yes,yes | yes,no | no,yes | no,no | yes,yes | yes,no | no,yes | no,no | yes,yes | yes,no | no,yes | no,no | yes,yes | yes,no | no,yes | no,no | yes,yes | yes,no | no,yes | no,no |
| Age |  |  |  |  |  |  |  |  |  |  |  |  |  |  |  |  |  |  |  |  |
| 18-26 | 754 (77.7) | 149 (15.4) | 45 (4.6) | 22 (2.3) | 656 (66.1) | 129 (13.0) | 87 (8.8) | 121 (12.2) | 550 (60.4) | 118 (13.0) | 65 (7.1) | 178 (19.5) | 511 (53.1) | 122 (12.7) | 91 (9.4) | 239 (24.8) | 439 (44.6) | 111 (11.3) | 106 (10.8) | 329 (33.4) |
| 27-35 | 958 (63.3) | 361 (23.8) | 105 (6.9) | 90 (5.9) | 817 (53.8) | 252 (16.6) | 138 (9.1) | 311 (20.5) | 775 (48.1) | 208 (12.9) | 157 (9.7) | 471 (29.2) | 569 (37.2) | 160 (10.5) | 171 (11.2) | 631 (41.2) | 489 (31.8) | 121 (7.9) | 125 (8.1) | 805 (52.3) |
| 36-45 | 401 (56.5) | 185 (26.1) | 54 (7.6) | 70 (9.9) | 351 (51.4) | 106 (15.5) | 53 (7.8) | 173 (25.3) | 310 (46.1) | 85 (12.6) | 58 (8.6) | 219 (32.6) | 225 (32.1) | 75 (10.7) | 72 (10.3) | 328 (46.9) | 190 (28.4) | 43 (6.4) | 50 (7.5) | 385 (57.6) |
| *P value* | *<0.001* | | | | *<0.001* | | | | *<0.001* | | | | *<0.001* | | | | *<0.001* | | | |
| Local GDP (100 million RMB) |  |  |  |  |  |  |  |  |  |  |  |  |  |  |  |  |  |  |  |  |
| ≤6000 | 712 (61.8) | 282 (24.5) | 89 (7.7) | 70 (6.1) | 618 (53.9) | 176 (15.3) | 109 (9.5) | 244 (21.3) | 529 (46.6) | 162 (14.3) | 110 (9.7) | 335 (29.5) | 405 (35.1) | 145 (12.6) | 125 (10.8) | 480 (41.6) | 367 (32.4) | 125 (11.0) | 93 (8.2) | 548 (48.4) |
| 6001-16000 | 469 (68.2) | 141 (20.5) | 41 (6.0) | 37 (5.4) | 427 (62.9) | 91 (13.4) | 53 (7.8) | 108 (15.9) | 370 (51.8) | 101 (14.1) | 50 (7.0) | 193 (27.0) | 288 (41.8) | 72 (10.4) | 82 (11.9) | 247 (35.8) | 250 (35.6) | 54 (7.7) | 59 (8.3) | 342 (48.4) |
| ≥16001 | 932 (68.9) | 272 (20.1) | 74 (5.5) | 75 (5.5) | 779 (56.9) | 220 (16.1) | 116 (8.5) | 253 (18.5) | 736 (54.8) | 148 (11.0) | 120 (8.9) | 340 (25.3) | 612 (45.3) | 140 (10.4) | 127 (9.4) | 471 (34.9) | 501 (36.9) | 96 (7.1) | 131 (9.7) | 629 (46.4) |
| *P value* | *0.009* | | | | *0.011* | | | | *0.001* | | | | *<0.001* | | | | *0.005* | | | |
| Local HDI |  |  |  |  |  |  |  |  |  |  |  |  |  |  |  |  |  |  |  |  |
| <0.7 | 36 (65.5) | 13 (23.6) | 4  (7.3) | 2  (3.6) | 35 (61.4) | 10 (17.5) | 4  (7.0) | 8 (14.0) | 16 (35.6) | 9 (20.0) | 6 (13.3) | 14 (31.1) | 16 (38.1) | 3  (7.1) | 5 (11.9) | 18 (42.9) | 18 (31.0) | 4  (6.9) | 8 (13.8) | 28 (48.3) |
| 0.7-0.8 | 1323 (65.2) | 464 (22.9) | 138 (6.8) | 103 (5.1) | 1163 (56.1) | 323 (15.6) | 194 (9.4) | 393 (19.0) | 970 (49.4) | 278 (14.2) | 179 (9.1) | 535 (27.3) | 792 (39.1) | 245 (12.1) | 213 (10.5) | 775 (38.3) | 687 (34.8) | 190 (9.6) | 180 (9.1) | 918 (46.5) |
| ≥0.8 | 754 (67.9) | 218 (19.6) | 62 (5.6) | 77 (6.9) | 626 (58.8) | 154 (14.5) | 80 (7.5) | 204 (19.2) | 649 (54.7) | 124 (10.4) | 95 (8.0) | 319 (26.9) | 497 (44.1) | 109 (9.7) | 116 (10.3) | 405 (35.9) | 413 (35.6) | 81 (7.0) | 93 (8.0) | 573 (49.4) |
| *P value* | *0.094* | | | | *0.475* | | | | *0.005* | | | | *0.108* | | | | *0.105* | | | |
| Ethnicity |  |  |  |  |  |  |  |  |  |  |  |  |  |  |  |  |  |  |  |  |
| Han Chinese | 1990 (66.9) | 631 (21.2) | 192 (6.5) | 162 (5.4) | 1718 (57.4) | 455 (15.2) | 259 (8.6) | 563 (18.8) | 1554 (52.1) | 375 (12.6) | 264 (8.9) | 787 (26.4) | 1219 (41.0) | 329 (11.1) | 312 (10.5) | 1112 (37.4) | 1050 (35.1) | 252 (8.4) | 267 (8.9) | 1423 (47.6) |
| Minority Chinese | 123 (56.2) | 64 (29.2) | 12 (5.5) | 20 (9.1) | 106 (53.3) | 32 (16.1) | 19 (9.5) | 42 (21.1) | 81 (37.9) | 36 (16.8) | 16 (7.5) | 81 (37.9) | 86 (38.7) | 28 (12.6) | 22 (9.9) | 86 (38.7) | 68 (33.8) | 23 (11.4) | 14 (7.0) | 96 (47.8) |
| *P value* | *0.002* | | | | *0.719* | | | | *<0.001* | | | | *0.833* | | | | *0.407* | | | |
| Educational level |  |  |  |  |  |  |  |  |  |  |  |  |  |  |  |  |  |  |  |  |
| Associate’s degree or below | 614 (68.5) | 188 (21.0) | 52 (5.8) | 43 (4.8) | 561 (60.1) | 137 (14.7) | 86 (9.2) | 149 (16.0) | 482 (53.2) | 123 (13.6) | 71 (7.8) | 230 (25.4) | 401 (43.9) | 110 (12.0) | 87 (9.5) | 316 (34.6) | 342 (37.1) | 105 (11.4) | 99 (10.7) | 376 (40.8) |
| Bachelor degree | 1435 (65.1) | 490 (22.2) | 146 (6.6) | 132 (6.0) | 1206 (55.8) | 329 (15.2) | 187 (8.6) | 440 (20.4) | 1100 (50.3) | 275 (12.6) | 202 (9.2) | 612 (28.0) | 860 (39.4) | 241 (11.0) | 237 (10.9) | 844 (38.7) | 733 (33.7) | 162 (7.5) | 174 (8.0) | 1103 (50.8) |
| Master degree or above | 64 (68.1) | 17 (18.1) | 6  (6.4) | 7  (7.4) | 57 (57.6) | 21 (21.2) | 5  (5.1) | 16 (16.2) | 53 (53.5) | 13 (13.1) | 7  (7.1) | 26 (26.3) | 44 (44.9) | 6  (6.1) | 10 (10.2) | 38 (38.8) | 43 (43.4) | 8  (8.1) | 8  (8.1) | 40 (40.4) |
| *P value* | *0.543* | | | | *0.036* | | | | *0.537* | | | | *0.106* | | | | *<0.001* | | | |
| Marital status |  |  |  |  |  |  |  |  |  |  |  |  |  |  |  |  |  |  |  |  |
| No partner | 585 (74.1) | 136 (17.2) | 43 (5.4) | 25 (3.2) | 524 (61.0) | 132 (15.4) | 83 (9.7) | 120 (14.0) | 475 (59.2) | 85 (10.6) | 69 (8.6) | 174 (21.7) | 411 (50.7) | 85 (10.5) | 87 (10.7) | 227 (28.0) | 330 (41.1) | 95 (11.8) | 94 (11.7) | 283 (35.3) |
| Not married but have partner | 338 (77.0) | 71 (16.2) | 19 (4.3) | 11 (2.5) | 282 (65.1) | 64 (14.8) | 35 (8.1) | 52 (12.0) | 229 (56.0) | 55 (13.4) | 33 (8.1) | 92 (22.5) | 218 (49.8) | 63 (14.4) | 48 (11.0) | 109 (24.9) | 191 (45.2) | 40 (9.5) | 42 (9.9) | 150 (35.5) |
| Married | 1190 (60.5) | 488 (24.8) | 142 (7.2) | 146 (7.4) | 1018 (53.5) | 291 (15.3) | 160 (8.4) | 433 (22.8) | 931 (47.0) | 271 (13.7) | 178 (9.0) | 602 (30.4) | 676 (34.7) | 209 (10.7) | 199 (10.2) | 862 (44.3) | 597 (30.3) | 140 (7.1) | 145 (7.4) | 1086 (55.2) |
| *P value* | *<0.001* | | | | *<0.001* | | | | *<0.001* | | | | *<0.001* | | | | *<0.001* | | | |
| Professional title |  |  |  |  |  |  |  |  |  |  |  |  |  |  |  |  |  |  |  |  |
| No title | 218 (76.2) | 47 (16.4) | 15 (5.2) | 6  (2.1) | 221 (69.7) | 35 (11.0) | 28 (8.8) | 33 (10.4) | 161 (59.0) | 36 (13.2) | 22 (8.1) | 54 (19.8) | 137 (47.1) | 40 (13.7) | 30 (10.3) | 84 (28.9) | 121 (39.9) | 32 (10.6) | 38 (12.5) | 112 (37.0) |
| Primary professional title | 1246 (68.5) | 384 (21.1) | 101 (5.6) | 88 (4.8) | 1034 (56.4) | 308 (16.8) | 168 (9.2) | 323 (17.6) | 971 (52.0) | 248 (13.3) | 161 (8.6) | 487 (26.1) | 803 (43.1) | 203 (10.9) | 200 (10.7) | 657 (35.3) | 663 (36.1) | 177 (9.6) | 162 (8.8) | 837 (45.5) |
| Middle professional title or above | 649 (59.6) | 264 (24.2) | 88 (8.1) | 88 (8.1) | 569 (54.5) | 144 (13.8) | 82 (7.9) | 249 (23.9) | 503 (47.7) | 127 (12.0) | 97 (9.2) | 327 (31.0) | 365 (35.1) | 114 (11.0) | 104 (10.0) | 457 (43.9) | 334 (31.8) | 66 (6.3) | 81 (7.7) | 570 (54.2) |
| *P value* | *<0.001* | | | | *<0.001* | | | | *0.004* | | | | *<0.001* | | | | *<0.001* | | | |
| Hospital level |  |  |  |  |  |  |  |  |  |  |  |  |  |  |  |  |  |  |  |  |
| Primary and secondary hospital | 512 (62.4) | 200 (24.4) | 60 (7.3) | 48 (5.9) | 472 (55.4) | 120 (14.1) | 75 (8.8) | 185 (21.7) | 431 (52.7) | 101 (12.3) | 71 (8.7) | 215 (26.3) | 328 (39.3) | 86 (10.3) | 96 (11.5) | 324 (38.8) | 257 (32.0) | 72 (9.0) | 71 (8.9) | 402 (50.1) |
| Tertiary hospital | 1601 (67.4) | 495 (20.9) | 144 (6.1) | 134 (5.6) | 1352 (57.7) | 367 (15.7) | 203 (8.7) | 420 (17.9) | 1204 (50.7) | 310 (13.0) | 209 (8.8) | 653 (27.5) | 977 (41.4) | 271 (11.5) | 238 (10.1) | 874 (37.0) | 861 (36.0) | 203 (8.5) | 210 (8.8) | 1117 (46.7) |
| *P value* | *0.061* | | | | *0.096* | | | | *0.790* | | | | *0.370* | | | | *0.228* | | | |
| Monthly income (CNY) |  |  |  |  |  |  |  |  |  |  |  |  |  |  |  |  |  |  |  |  |
| ≤4000 | 864 (62.0) | 341 (24.5) | 103 (7.4) | 85 (6.1) | 826 (54.3) | 217 (14.3) | 141 (9.3) | 336 (22.1) | 675 (47.6) | 193 (13.6) | 132 (9.3) | 419 (29.5) | 552 (37.0) | 156 (10.4) | 159 (10.6) | 626 (41.9) | 455 (30.3) | 130 (8.6) | 130 (8.6) | 788 (52.4) |
| 4001-8000 | 815 (67.5) | 256 (21.2) | 78 (6.5) | 58 (4.8) | 685 (59.0) | 189 (16.3) | 96 (8.3) | 191 (16.5) | 618 (52.2) | 156 (13.2) | 102 (8.6) | 309 (26.1) | 481 (43.1) | 132 (11.8) | 125 (11.2) | 378 (33.9) | 445 (39.1) | 105 (9.2) | 101 (8.9) | 488 (42.8) |
| ≥8001 | 434 (73.1) | 98 (16.5) | 23 (3.9) | 39 (6.6) | 313 (61.0) | 81 (15.8) | 41 (8.0) | 78 (15.2) | 342 (58.0) | 62 (10.5) | 46 (7.8) | 140 (23.7) | 272 (46.5) | 69 (11.8) | 50 (8.5) | 194 (33.2) | 218 (39.6) | 40 (7.3) | 50 (9.1) | 243 (44.1) |
| *P value* | *<0.001* | | | | *0.001* | | | | *0.003* | | | | *<0.001* | | | | *<0.001* | | | |
| Number of children |  |  |  |  |  |  |  |  |  |  |  |  |  |  |  |  |  |  |  |  |
| No child | 1078 (74.9) | 248 (17.2) | 65 (4.5) | 48 (3.3) | 923 (62.5) | 231 (15.6) | 131 (8.9) | 192 (13.0) | 817 (58.1) | 172 (12.2) | 118 (8.4) | 299 (21.3) | 709 (49.5) | 167 (11.7) | 156 (10.9) | 400 (27.9) | 614 (42.4) | 151 (10.4) | 158 (10.9) | 523 (36.2) |
| Have one child | 757 (61.4) | 292 (23.7) | 97 (7.9) | 86 (7.0) | 629 (52.8) | 183 (15.4) | 92 (7.7) | 288 (24.2) | 599 (47.0) | 167 (13.1) | 111 (8.7) | 398 (31.2) | 444 (34.7) | 127 (9.9) | 133 (10.4) | 577 (45.0) | 362 (28.8) | 87 (6.9) | 90 (7.2) | 717 (57.1) |
| Have two or more children | 278 (53.2) | 155 (29.6) | 42 (8.0) | 48 (9.2) | 272 (51.8) | 73 (13.9) | 55 (10.5) | 125 (23.8) | 219 (42.7) | 72 (14.0) | 51 (9.9) | 171 (33.3) | 152 (31.6) | 63 (13.1) | 45 (9.4) | 221 (45.9) | 142 (28.9) | 37 (7.5) | 33 (6.7) | 279 (56.8) |
| *P value* | *<0.001* | | | | *<0.001* | | | | *<0.001* | | | | *<0.001* | | | | *<0.001* | | | |
| Vaccination behavior |  |  |  |  |  |  |  |  |  |  |  |  |  |  |  |  |  |  |  |  |
| Not intended to vaccinate | 54 (24.9) | 51 (23.5) | 37 (17.1) | 75 (34.6) | 45 (22.5) | 22 (11.0) | 15 (7.5) | 118 (59.0) | 44 (20.6) | 16 (7.5) | 17 (7.9) | 137 (64.0) | 31 (13.2) | 13 (5.5) | 13 (5.5) | 178 (75.7) | 31 (12.3) | 10 (4.0) | 10 (4.0) | 202 (79.8) |
| Have not been vaccinated or made an appointment | 1353 (66.7) | 453 (22.3) | 127 (6.3) | 96 (4.7) | 1179 (57.5) | 322 (15.7) | 178 (8.7) | 373 (18.2) | 1018 (50.1) | 264 (13.0) | 195 (9.6) | 553 (27.2) | 766 (38.5) | 225 (11.3) | 209 (10.5) | 790 (39.7) | 640 (32.6) | 166 (8.4) | 182 (9.3) | 977 (49.7) |
| Have been vaccinated or have made an appointment | 706 (74.5) | 191 (20.1) | 40 (4.2) | 11 (1.2) | 600 (63.7) | 143 (15.2) | 85 (9.0) | 114 (12.1) | 573 (60.3) | 131 (13.8) | 68 (7.2) | 178 (18.7) | 508 (52.4) | 119 (12.3) | 112 (11.6) | 230 (23.7) | 447 (45.8) | 99 (10.2) | 89 (9.1) | 340 (34.9) |
| *P value* | *<0.001* | | | | *<0.001* | | | | *<0.001* | | | | *<0.001* | | | | *<0.001* | | | |

**Supplementary Table 2. Attitude shift towards HPV vaccines between two payment scenarios across sociodemographics**

|  | Respondents remaining with cheaper vaccine regardless of payment scenario (%) | Respondents choosing more expensive vaccine if partly covered by governmental subsidy (instead of basic medical insurance) (%) | Respondents moving from more expensive to cheaper vaccine if partly covered by governmental subsidy (instead of basic medical insurance) (%) | Respondents remaining with more expensive vaccine, regardless of payment scenario (%) | *P value* |
| --- | --- | --- | --- | --- | --- |
| Overall | 6222 (39.0) | 1148 (7.2) | 433 (2.7) | 8166 (51.1) |  |
| Age |  |  |  |  |  |
| 18-26 | 1247 (25.9) | 310 (6.4) | 101 (2.1) | 3164 (65.6) | *<0.001* |
| 27-35 | 3395 (44.0) | 569 (7.4) | 224 (2.9) | 3526 (45.7) |  |
| 36-45 | 1580 (46.0) | 269 (7.8) | 108 (3.1) | 1476 (43.0) |  |
| Local GDP (100 million RMB) |  |  |  |  |  |
| ≤6000 | 2494 (43.6) | 449 (7.8) | 177 (3.1) | 2604 (45.5) | *<0.001* |
| 6001-16000 | 1314 (37.8) | 263 (7.6) | 93 (2.7) | 1803 (51.9) |  |
| ≥16001 | 2414 (35.6) | 436 (6.4) | 163 (2.4) | 3759 (55.5) |  |
| Local HDI |  |  |  |  |  |
| <0.7 | 99 (38.5) | 20 (7.8) | 12 (4.7) | 126 (49.0) | *<0.001* |
| 0.7-0.8 | 4109 (40.8) | 772 (7.7) | 288 (2.9) | 4894 (48.6) |  |
| ≥0.8 | 2014 (35.7) | 356 (6.3) | 133 (2.4) | 3146 (55.7) |  |
| Ethnicity |  |  |  |  |  |
| Han Chinese | 5712 (38.3) | 1065 (7.1) | 391 (2.6) | 7746 (51.9) | *<0.001* |
| Minority Chinese | 510 (48.3) | 83 (7.9) | 42 (4.0) | 420 (39.8) |  |
| Educational level |  |  |  |  |  |
| Associate’s degree or below | 1629 (35.6) | 302 (6.6) | 115 (2.5) | 2526 (55.2) | *<0.001* |
| Bachelor degree | 4414 (40.5) | 817 (7.5) | 303 (2.8) | 5374 (49.3) |  |
| Master degree or above | 179 (36.6) | 29 (5.9) | 15 (3.1) | 266 (54.4) |  |
| Marital status |  |  |  |  |  |
| No partner | 1221 (30.1) | 253 (6.2) | 91 (2.2) | 2498 (61.5) | *<0.001* |
| Not married but have partner | 594 (27.7) | 153 (7.1) | 61 (2.8) | 1334 (62.3) |  |
| Married | 4407 (45.1) | 742 (7.6) | 281 (2.9) | 4334 (44.4) |  |
| Professional title |  |  |  |  |  |
| No title | 407 (27.7) | 93 (6.3) | 30 (2.0) | 940 (63.9) | *<0.001* |
| Primary professional title | 3426 (37.2) | 649 (7.0) | 243 (2.6) | 4903 (53.2) |  |
| Middle professional title or above | 2389 (45.3) | 406 (7.7) | 160 (3.0) | 2323 (44.0) |  |
| Hospital level |  |  |  |  |  |
| Primary and secondary hospital | 1708 (41.4) | 281 (6.8) | 100 (2.4) | 2037 (49.4) | *0.002* |
| Tertiary hospital | 4514 (38.1) | 867 (7.3) | 333 (2.8) | 6129 (51.8) |  |
| Monthly income (CNY) |  |  |  |  |  |
| ≤4000 | 2897 (39.5) | 579 (7.9) | 180 (2.5) | 3672 (50.1) | *0.001* |
| 4001-8000 | 2217 (38.2) | 411 (7.1) | 168 (2.9) | 3012 (51.9) |  |
| ≥8001 | 1108 (39.1) | 158 (5.6) | 85 (3.0) | 1482 (52.3) |  |
| Number of children |  |  |  |  |  |
| No child | 2132 (29.6) | 483 (6.7) | 171 (2.4) | 4414 (61.3) | *<0.001* |
| Have one child | 2807 (45.0) | 449 (7.2) | 186 (3.0) | 2794 (44.8) |  |
| Have two or more children | 1283 (50.7) | 216 (8.5) | 76 (3.0) | 958 (37.8) |  |
